# Artificial intelligence enables comprehensive genome interpretation and nomination of candidate diagnoses for rare genetic diseases

**DOI:** 10.1186/s13073-021-00965-0

**Published:** 2021-10-14

**Authors:** Francisco M. De La Vega, Shimul Chowdhury, Barry Moore, Erwin Frise, Jeanette McCarthy, Edgar Javier Hernandez, Terence Wong, Kiely James, Lucia Guidugli, Pankaj B. Agrawal, Casie A. Genetti, Catherine A. Brownstein, Alan H. Beggs, Britt-Sabina Löscher, Andre Franke, Braden Boone, Shawn E. Levy, Katrin Õunap, Sander Pajusalu, Matt Huentelman, Keri Ramsey, Marcus Naymik, Vinodh Narayanan, Narayanan Veeraraghavan, Paul Billings, Martin G. Reese, Mark Yandell, Stephen F. Kingsmore

**Affiliations:** 1 Fabric Genomics Inc., Oakland, CA USA; 2 Department of Biomedical Data Science, Stanford University School of Medicine, Stanford, CA USA; 3 Current Address: Tempus Labs Inc., Redwood City, CA 94065 USA; 4 Rady Children’s Institute for Genomic Medicine, San Diego, CA USA; 5 Department of Human Genetics, Utah Center for Genetic Discovery, University of Utah, Salt Lake City, UT USA; 6 Division of Genetics and Genomics, The Manton Center for Orphan Disease Research, Boston Children’s Hospital, Harvard Medical School, Boston, MA USA; 7 Division of Newborn Medicine, Boston Children’s Hospital, Boston, MA USA; 8 Institute of Clinical Molecular Biology, Christian-Albrechts-University of Kiel & University Hospital Schleswig-Holstein, Kiel, Germany; 9 HudsonAlpha Institute for Biotechnology, Huntsville, AL USA; 10 Department of Clinical Genetics, United Laboratories, Tartu University Hospital, Tartu, Estonia; 11 Department of Clinical Genetics, Institute of Clinical Medicine, University of Tartu, Tartu, Estonia; 12 Center for Rare Childhood Disorders, Translational Genomics Research Institute, Phoenix, AZ USA

## Abstract

**Background:**

Clinical interpretation of genetic variants in the context of the patient’s phenotype is becoming the largest component of cost and time expenditure for genome-based diagnosis of rare genetic diseases. Artificial intelligence (AI) holds promise to greatly simplify and speed genome interpretation by integrating predictive methods with the growing knowledge of genetic disease. Here we assess the diagnostic performance of Fabric GEM, a new, AI-based, clinical decision support tool for expediting genome interpretation.

**Methods:**

We benchmarked GEM in a retrospective cohort of 119 probands, mostly NICU infants, diagnosed with rare genetic diseases, who received whole-genome or whole-exome sequencing (WGS, WES). We replicated our analyses in a separate cohort of 60 cases collected from five academic medical centers. For comparison, we also analyzed these cases with current state-of-the-art variant prioritization tools. Included in the comparisons were trio, duo, and singleton cases. Variants underpinning diagnoses spanned diverse modes of inheritance and types, including structural variants (SVs). Patient phenotypes were extracted from clinical notes by two means: manually and using an automated clinical natural language processing (CNLP) tool. Finally, 14 previously unsolved cases were reanalyzed.

**Results:**

GEM ranked over 90% of the causal genes among the top or second candidate and prioritized for review a median of 3 candidate genes per case, using either manually curated or CNLP-derived phenotype descriptions. Ranking of trios and duos was unchanged when analyzed as singletons. In 17 of 20 cases with diagnostic SVs, GEM identified the causal SVs as the top candidate and in 19/20 within the top five, irrespective of whether SV calls were provided or inferred ab initio by GEM using its own internal SV detection algorithm. GEM showed similar performance in absence of parental genotypes. Analysis of 14 previously unsolved cases resulted in a novel finding for one case, candidates ultimately not advanced upon manual review for 3 cases, and no new findings for 10 cases.

**Conclusions:**

GEM enabled diagnostic interpretation inclusive of all variant types through automated nomination of a very short list of candidate genes and disorders for final review and reporting. In combination with deep phenotyping by CNLP, GEM enables substantial automation of genetic disease diagnosis, potentially decreasing cost and expediting case review.

**Supplementary Information:**

The online version contains supplementary material available at 10.1186/s13073-021-00965-0.

## Background

A central tenet of genomic medicine is that outcomes are improved when symptom-based diagnoses and treatments are augmented with genetic diagnoses and genotype-differentiated treatments. Worldwide, an estimated 7 million infants are born with serious genetic disorders every year [1]. The last decade witnessed a huge increase in the catalog of genes associated with Mendelian conditions, from about 2300 in 2010 [2], to over 6700 by the end of 2020 [3]. The translation of that knowledge, in conjunction with major improvements in WES and WGS and downstream analytical pipelines, has enabled increased rates of diagnosis, from about 10%, with single gene tests, to over 50% [4]. While limitations of read alignment and variant calling were major obstacles to early clinical implementations of WES and WGS [5], they have been largely removed by algorithmic advances, hardware acceleration, and parallelization through cloud computing [6, 7]. However, clinical interpretation of genetic variants in the context of the patient’s phenotype remains largely manual and extremely labor-intensive, requiring highly trained expert input. This remains a major barrier to widespread adoption and contributes to continued low rates of genomic testing for patients with suspected genetic disorders despite strong evidence for diagnostic and clinical utility and cost effectiveness [8].

The major challenge for genome-based diagnosis of rare genetic disease is to identify a putative disease-causing variant amid approximately four million benign variants in each genome, a problem akin to finding a needle in a haystack [9]. Clinical genome interpretation is, by necessity, performed by highly trained, scarce, genome analysts, genetic counselors, and laboratory directors [10]. For an average of 100 variants for review per case [11], this translates to 50–100 h of expert review per patient [10]. In practice, this has led to review of only about 10 variants per case, which somewhat defeats the purpose of genome-wide sequencing.

The genome interpretation process consists of iterative variant filtering, coupled with evidence-based review of candidate disease-causing variants [12]. This process was almost entirely manual until the advent of variant prioritization algorithms, such as Annovar [13] and VAAST [14], and was later improved by the integration of patient phenotypes in analyses, e.g., Phevor [15], Exomiser [16], Phen-Gen [17], Phenolyzer [18], and more recently Amelie [19]. While these tools accelerate review times, their stand-alone performance has been insufficient for widespread clinical adoption, in part due to their inability to appropriately interpret structural variants (SVs). SVs account for over 10% of Mendelian disease [20, 21], and about 20% of diagnoses in routine neonatal intensive care unit (NICU) [22] and pediatric patients [23]. Unified methods for prioritization of SVs, SNVs, and small indels are a fundamental requirement for further automation of genome interpretation.

The use of artificial intelligence (AI) has made significant inroads in healthcare [24], and a new class of genome interpretation methods [19, 25–28] are being developed with the promise of removing the interpretation bottleneck for rare genetic disease diagnosis through electronic clinical decision support systems (eCDSS) [29]. Speed and accuracy of interpretation are particularly important for seriously ill children in the NICU [27], where diagnosis in the first 24–48 h of life has been shown to maximally improve health outcomes [30]. The settings and extent to which AI facilitates diagnosis are still being investigated [27, 28]. Issues include what types of AI methods are most suitable (e.g., Bayesian networks, decision trees, neural nets [31]); how they compare with current variant prioritization approaches in terms of accuracy; their diagnostic performance across different clinical scenarios and variant types; their potential to offer new forms of decision support; and how well they integrate with automated patient phenotyping and clinical decision making [27, 28, 32].

Algorithmic benchmarking in this domain is no simple matter. Hitherto, most attempts have used simulated cases (created by adding known disease-causing variants to reference exomes and genomes), included only a few cases, derived from a single center, or were limited to certain variant types [17, 33, 34]. Such benchmarking is inherently limited, as it is not representative of the true diversity of genetic diseases and variant types (e.g., by omitting cases with causal SVs), and provide no means to evaluate the impact of different sequencing and variant calling pipelines on performance.

Here we describe and benchmark the diagnostic performance of Fabric GEM (hereafter referred to as “GEM”), a new AI-based eCDSS, and compare it to current variant prioritization approaches using a diverse cohort of retrospective pediatric cases from the Rady Children’s Institute for Genomic Medicine (RCIGM). These cases are largely comprised of seriously ill NICU infants; all were diagnosed with Mendelian conditions following WGS (or, in a few cases, WES), using a combination of filtering and variant prioritization approaches. These real-world cases encompass the breadth of phenotypes and disease-causing variants, including pathogenic SVs. We then sought to replicate the diagnostic performance of GEM in a second set of affected, diagnosed, and undiagnosed children outside the NICU. They were collected from five different academic medical centers, mostly consisting of WES, to examine the generalizability of GEM’s diagnostic performance to other sequencing, variant calling pipelines, and clinical settings. Finally, we reanalyzed a set of previously negative RCIGM cases to evaluate the ability of GEM to identify new diagnoses without suggesting numerous false positives that would lead to time-consuming case reviews. Our results show that rapid, accurate, and comprehensive WGS- and WES-based diagnosis is achievable through integration of new data modalities with algorithmic innovations made possible by AI.

## Methods

### Patient selection, phenotyping, and specimen sequencing

This retrospective study was designed to provide benchmark data to test the GEM eCDSS. We compiled 119 cases from Rady Children’s Hospital (the *Benchmark* cohort), consisting of mostly NICU admissions, and 60 additional cases from five academic medical centers (the *Validation* cohort), which consisted mostly of referrals from genetic clinics and none included causal structural variants, as described below.

#### Rady Children’s Hospital

In total, 119 cases with primary findings, deemed definitively solved using previously published methods [27, 30, 35], and 14 negative cases, were sequenced as part of the rapid-WGS (rWGS) sequencing program at the Rady Children’s Hospital Clinical Genome Center. These cases where a sample of convenience, drawn from the first symptomatic children who were enrolled in four previously published studies that examined the diagnostic rate, time to diagnosis, clinical utility of diagnosis, outcomes, and health care utilization of rWGS between 26 July 2016 and 25 September 2018 at Rady Children’s Hospital, San Diego, USA. One of the studies was a randomized controlled trial of genome and exome sequencing (ClinicalTrials.gov identifier: NCT03211039) [30]; the others were cohort studies (ClinicalTrials.gov identifiers: NCT02917460, and NCT03385876) [35–40]. All subjects had a symptomatic illness of unknown etiology in which a genetic disorder was suspected, had a Rady Children’s Hospital Epic EHR, and that had clinical phenotype descriptions expressed as human phenotype ontology terms both manually curated by clinicians and automatically extracted by CNLP (Additional file 1: Table S1).

WGS (or in a few instances WES) was performed as previously described [35, 40]. Briefly, PCR-free WGS was performed to an average of 40× coverage in the Illumina HiSeq 2000, HiSeq 4000, and NovaSeq 6000 sequencers. Alignment and sequence variant calling were performed using the Illumina DRAGEN software, while copy number variation was identified through an approach that integrates the tools Manta [41] and CNVnator [42]. Structural variants were then filtered for recurrent artifacts observed in previous non-affected cases and only included in the input VCF file if they overlap a known disease gene (OMIM). All variants reported as primary findings were validated orthogonally by Sanger sequencing. In the case of trios, de novo origin of reported variants was established by comparing to their parents’ data. In some older cases, SV calling was not performed; any causal SVs therein were identified by an orthogonal CGH microarray or manual inspection of alignments. In what follows, we refer to these 119 cases with primary findings as the *Benchmark* cohort, and the 14 negative cases as the *Unsolved* cohort.

#### Boston Children’s Hospital

Eleven cases (all single probands) from the Beggs Lab, Congenital Myopathy Research Program laboratory, and Manton Center for Orphan Disease Research at Boston Children’s Hospital were included in the analysis [43–48].

Libraries (TruSeq DNA v2 Sample Preparation kit; Illumina, San Diego, CA) and whole-exome capture (EZ Exome 2.0, Roche) were performed according to manufacturer protocols from DNA extracted from blood samples. WES was carried out on an Illumina HiSeq 2000. Reads were aligned to the GRCh37/hg19 human genome assembly using an in-house assembler. Variants were called using Gene Analysis Toolkit (GATK) version 3.1 or higher (Broad Institute, Cambridge, MA) and were Sanger confirmed by the Boston Children’s Hospital IDDRC Molecular Genetics Core Facility.

#### Christian-Albrechts University of Kiel

Twelve cases (all single probands) from the Institute of Clinical Molecular Biology (IKMB) were included in the analysis [49–55].

Illumina’s Nextera/TruSeq whole-exome target capture method was applied. WES was carried out on the Illumina HiSeq/NovaSeq platforms. Reads were aligned to the GRCh37/hg19 human genome assembly using BWA-MEM version 0.7.17 and variants called using GATK version 4.1.6.0 (Broad Institute, Cambridge, MA).

#### HudsonAlpha Institute for Biotechnology

Three cases (two trios and a single proband) from the Clinical Services Laboratory at HudsonAlpha Institute for Biotechnology, including cases from the Clinical Sequencing Evidence-Generating Research (CSER) consortium, were included in the analysis [56–59].

WGS was carried out on an Illumina HiSeq X. Reads were aligned to the GRCh37/hg19 human genome assembly followed by variant calling using the Illumina DRAGEN software version 3.2.8 (Illumina, Inc. San Diego, CA).

#### Translational Genomics Research Institute

Twenty-three cases (including singletons, duos, trios, and quads) from the Center for Rare Childhood Disorders at The Translational Genomics Research Institute (TGen) were included in the analysis [60, 61].

WES or WGS sequencing was carried out on an Illumina HiSeq 2000, HiSeq 2500, HiSeq 4000, or NovaSeq6000. For WES, the Agilent SureSelect Human All Exon V6 or CRE V2 target capture method was applied. Reads were aligned to reference GRCh37 version hs37d5 and variants called using GATK Haplotype caller version 3.3-0-g37228af (Broad Institute, Cambridge, MA).

#### Tartu University Hospital

Eleven cases from Tartu University Hospital in Estonia that had undergone WES were included in the analysis [62–64].

Nextera Rapid Capture Exome Kit-i (Illumina Inc.) target capture method was applied. WES was carried out on an Illumina HiSeq2500 sequencer. Reads were aligned to the GRCh37/hg19 human genome assembly using BWA-MEM version 0.5.9 and variants called using GATK Haplotype caller version 3.4 (Broad Institute, Cambridge, MA).

### Variant annotation and data sources

All analyses were performed based on the GRCh37 human genome assembly. Variant consequences and annotations were obtained with VEP v.95 [65] utilizing ENSEMBL transcripts version 95 (excluding non-coding transcripts) and selecting the canonical transcript for analysis. Transcript-specific prediction for evaluating variant deleteriousness was calculated with VVP [66], which were also used as input for VAAST [14]. Variants were annotated with ClinVar (version 20200419) [67] ensuring exact position and base match. Gene conditions were extracted from OMIM (version 2020_07) [68] and HPO (obo file dated 2020-08-11) [69]. Gene symbols were harmonized using ENSEMBL and HGNC databases controlling for synonymous gene symbols.

### AI-based disease gene and condition prioritization

AI-based prioritization and scoring of candidate disease genes and diagnostic conditions was performed using Fabric GEM [70], which is a commercially available eCDSS part of the Fabric Enterprise platform (Fabric Genomics, Oakland, CA). GEM inputs are genetic variant calls in VCF format and case metadata, including (optional) parental affection status, and patient (proband) phenotypes in the form of Human Phenotype Ontology (HPO) terms. The VCF files can include “small variants” (single nucleotide, multiple nucleotide, and small insertion/deletion variants), and (optionally) structural variants (insertion/deletions of over 50 bp, inversions, and/or copy number variants with imprecise ends). This information can be provided via an application programming interface or manually in the user interface. Data analysis is typically carried out in minutes depending on inputs. GEM outputs are displayed in an interactive report (Additional file 2: Figure S1) that includes a list of candidate genes ranked by the GEM gene score (see below), detailed information of patient variants present in each candidate gene, and conditions associated with each candidate gene ranked by GEM’s condition match (CM) score (explained below).

GEM aggregates inputs from multiple variant prioritization algorithms with genomic and clinical database annotations, using Bayesian means to score and prioritize potentially damaged genes and candidate diseases. Briefly, the algorithm parametrizes itself using the proband’s called variants as one-time, run-time training data, inferring the states of multiple variables directly from the input variant distribution, e.g., sex. Additional static training parameters were derived from the 1000 Genomes Project [71] and CEPH [72] genome datasets. GEM reevaluates genotype calls and quality scores considering read support, genomic location, proband sex, and potentially overlapping SVs, augmenting the genotype calls with more nuanced posterior probabilities, computing ploidy for each variant. GEM also computes the likelihood that the proband belongs to any of several different ancestry groups using the input genotypes together with gnomAD sub-population variant frequency data [73]. The probabilities of other, internal, variables, conditioned on each state (sex and ancestry, etc.) are then obtained using naive Bayes, controlling for non-independence of variables by calculating a correlation matrix at run time using the proband’s data. For example, after conditioning variant scores on ancestry, known inheritance pattern for the gene in question, gene location, and proband sex, GEM may conclude that a de novo variant is unlikely to participate in a disease-causing genotype, even though it is predicted to be highly damaging. Thus, highly damaging and de novo variants, even frameshifting ones, do not automatically receive high GEM scores. GEM uses the same procedure to evaluate and score biallelic genotypes for known and novel disease-gene candidates. The only difference is that the global prior (e.g., relative proportion of known disease genes with autosomal recessive vs. autosomal dominant inheritance patterns), rather than OMIM and HPO support for a particular inheritance pattern at that locus, is used to evaluate possible biallelic cases in novel gene candidates.

GEM’s gene scores are Bayes factors (BF) [74]. Analogous to the likelihood ratio test, a Bayes factor presents the log_10_ ratio between the posterior probabilities of two models, summarizing the relative support for the hypothesis (in this case) that the prioritized genotype damages the gene in which it resides and that explains the proband’s phenotype versus the contrapositional hypothesis that the variant neither damages the gene nor explains the proband’s phenotype. In keeping with established best practice [74], a log_10_ Bayes factor between 0 and 0.69 is considered moderate support, between 0.69 and 1.0 substantial support, between 1.0 and 2.0 strong support, and above 2.0, decisive support. A score less than 0 indicates that the counter hypothesis is more likely. For calculating the Bayes posterior p(M|D), the probability of the data given a model (pD|M) is derived from GEM’s severity scoring protocol, which includes input from the VAAST and VVP algorithms, and any available prior variant classifications from the ClinVar database. This model is conditioned upon sex, ancestry, feasible inheritance model, gene location, and gene-phenotype priors derived by seeding the provided patient HPO terms to the HPO ontology graph and subsequently obtaining priors for all genes in the HPO and GO ontologies by belief propagation using Phevor’s previously described Bayesian network-based algorithm [15]. The prior probability for the model (pM) is based upon known disease associations in the Mendelian conditions databases OMIM and/or HPO with the gene in question.

GEM’s Bayes factor-based scoring system is designed for ease of explanation and to speed interpretation. GEM scores are not intended to be definitive, rather they are designed to provide guidance for succinct case reviews carried out by clinical geneticists. Thus, GEM outputs also include several additional scores that provide additional guidance and improve explainability. GEM gene scores, for example, are accompanied by VAAST [14], VVP [66], and Phevor [15] posterior probabilities, conditioned upon the potentially confounding variables of proband sex, gene location, and ancestry, together with common variant genomic and clinical annotations (Additional file 2: Figure S1). These scores further ease interpretation, as they allow users to assess the major drivers of a GEM score and their relative contributions to it.

GEM also provides means to assess the Mendelian conditions associated with putative disease-causing genes as possible diagnoses via its condition match (CM) scores. Like gene scores, CM scores are Bayes factors and are derived from the log_10_ ratio of the posterior probability that HPO phenotype associations for a given Mendelian condition’s HPO are consistent with the proband’s phenotype versus the contrapositional hypothesis. For these calculations, the probability of the data, p(D|M), is determined using Phevor’s Bayesian algorithm to obtain a probability for each disease, conditioned upon the proband’s phenotype. The prior probability for the model, p(M), is the probability that one or more genes associated with the Mendelian condition (as documented in OMIM and/or HPO) contain a damaging genotype as ascertained by GEM’s severity scoring protocol. Condition match scores are displayed alongside each gene-associated condition for review (Additional file 2: Figure S7).

### Structural variant scoring and ab initio inference by GEM

At run time, GEM infers ab initio the existence of SVs, their coordinates, and their copy numbers (ploidy) in a probabilistic fashion using SNVs, sort indel calls, read depths, zygosity, and gnomAD frequency data. GEM searches the proband’s genotypes for evidence of three types of SV: deletions, duplications, and CNVs. Regions exhibiting loss of heterozygosity (LOH), for example, are used as evidence for heterozygous deletions. Genomic spans lacking expected variants, the signature of homozygous deletions, are identified using gnomAD population frequencies [73] to derive point estates that a given gnomAD variant would or would not be ascertained given its population frequency. Further evidence for duplications and deletions is derived from read support, e.g., approximately integer increases or decreases in depth across a region provide support for copy number variation. Point estimates at each site of a small variant call are further conditioned upon provided variables, such as genotype qualities, and inferred ones, such as sex and ancestry, to obtain more refined estimates. High scoring segments and their maximum likelihood start and end coordinates are identified using a Markov model [75]. The results are used to determine the degree of support for external SV calls, and as the basis for GEM’s own SV calls. For ease of reporting, ab initio SV calls that overlap an external SV call (default minimum reciprocal overlap of 33%) are replaced in the output by the external SV call as long as they still overlap the relevant scored genes.

### Benchmarking variant prioritization with VAAST, Phevor, and Exomiser

We used the Snakemake software [76] to create a workflow that analyzes cases with the VAAST, Phevor, and Exomiser algorithms. This workflow was only applied to the benchmark cohort to enable us to compare the performance of four genome interpretation tools with exactly the same inputs and annotations. The pipeline starts with a VCF file, family structure, affection status, and HPO terms and concludes with the outputs for each of the algorithms. VVP scores were obtained as described above and provided to VAAST as input. VAAST was provided pedigree information and affection status and was run in both dominant and recessive modes with results aggregated. Gene ranks for VAAST are reported for the highest scoring occurrence of the gene from aggregated outputs. Phevor was provided with HPO terms and VAAST scores as inputs. Ranks were selected as described for VAAST.

Exomiser [16] benchmark analyses were run with the same configuration used in the 100,000 Genomes Project [77], specifically (1) using the GRCh37 genome assembly; (2) analyzing autosomal and X-linked forms of dominant and recessive inheritance; (3) allele frequency sources from the 1000 Genomes Project [78], TopMed [79], UK10K [80], ESP, ExAC [81], and gnomAD [73] (except Ashkenazi Jewish); (4) pathogenicity sources from REVEL [82] and MVP [83]; and (5) including the steps failedVariantFilter, variantEffectFilter (remove non-coding variants), frequencyFilter with maxFrequency = 2.0, pathogenicityFilter with keepNonPathogenic = true, inheritanceFilter, omimPrioritiser, and hiPhivePrioritiser.

Exomiser was considered to have identified the diagnosed gene when it was scored as a candidate for any of the utilized modes of inheritance. None of the tools in this analysis were provided a target mode of inheritance (as it is unknown), and so the diagnostic gene rank for Exomiser was determined from its rank within the combined gene candidate list from all modes of inheritance (i.e., the same procedure used for VAAST and PHEVOR). The ranks within the combined list of candidate genes were generated by sorting gene-level candidates from all modes of inheritance on the Exomiser combinedScore in descending order with each candidate gene only added to the list on its first, highest scoring occurrence. Exomiser variant level scoring was not considered for determining candidates or ranking. All Exomiser analyses on the benchmark cohort ran to completion and successfully produced output; however, in 18 cases, Exomiser did not identify the true positive diagnostic gene as a scored candidate (i.e., it was absent from its output). A similar phenomenon was observed in 4 cases using VAAST. For both tools, these cases were considered false negatives.

### Impact of deep phenotypes derived from clinical NLP

The utility of HPO terms was investigated by rerunning all analyses from the benchmark cohort with three sets of HPO terms. The motivations for these analyses were first to determine how sensitive GEM is to phenotyping errors; and second, to compare the utility of CNLP-derived descriptions to manual ones. For each case, an HPO terms list was provided that included HPO terms manually curated by the analysis team when the case was originally solved. A second set of HPO terms was generated from NLP analysis of clinical notes related to the case using the CLiX ENRICH software (Clinithink, Alpharetta, GA) [28]. A randomized set of HPO terms was generated for each case whereby the number of HPO terms from the CliniThink analysis case was held constant, and alternate terms were randomly selected from the entire corpus of HPO terms across all samples with each selection probability determined by the number of times that term occurred in the corpus.

## Results and discussion

### GEM AI outperforms variant prioritization approaches

We benchmarked GEM, an AI-based eCDSS, using a cohort of 119 pediatric retrospective cases from Rady Children’s Institute for Genomic Medicine (RCIGM; benchmark cohort). Most of these were critically ill NICU infants who received genomic sequencing for diagnosis of genetic diseases. All had been diagnosed with one or more Mendelian conditions using a combination of manual filtering and variant prioritization approaches (“Methods”). To further validate performance, we also analyzed a second cohort comprised of 60 non-NICU, rare disease patients from five different academic medical centers (validation cohort). Finally, we reanalyzed a set of 14 previously analyzed probands that had remained undiagnosed by WGS. Our goal was to evaluate the ability of GEM to identify new diagnoses in these previously unsolved cases, without providing false positive findings that would result in time-consuming case reviews. To provide context for our performance benchmarks, we also ran three commonly used variant prioritization tools: VAAST [14], Phevor [15], and Exomiser [16].

The benchmark and validation cohorts included singleton probands, parent-offspring trios, different modes of inheritance, and both small causal variants (SNVs, and small insertions or deletions, indels; Table 1; Additional file 1: Table S1) and large structural variants (SV), some of which were causative (Table 2). In these retrospective analyses, we considered the variants, disease genes, and conditions that were included as primary findings in the clinical report as the “gold standard” truth set.

**Table 1:** Characteristics of case cohorts. Benchmark cohort, 119 cases total. Validation cohort, 60 cases total. Grand total, 179 cases

	Assay type		Variant type		Proband sex		Pedigree Type		
Mode of inheritance	WGS	WES	SNV/Indel	SV	Male	Female	Single	Duos	Trios
*Benchmark cohort*									
Autosomal dominant	70	11	66	15	36	45	35	6	40
Autosomal recessive	27	–	23	4	14	13	9	1	17
X-linked dominant	6	–	5	1	1	5	2	–	4
X-linked recessive	5	–	5	–	5	–	2	1	2
Sub-totals	108	11	99	20	56	63	48	8	63
*Validation cohort*									
Autosomal dominant	3	34	37	–	10	27	15	2	20
Autosomal recessive	1	14	15	–	5	10	9	–	6
X-linked dominant	1	5	6	–	3	3	1	3	2
X-linked recessive	–	2	2	–	2	–	1	–	1
Sub-totals	5	55	60	0	20	40	26	5	29

**Table 2:** Diagnostic structural variants identified by GEM in the benchmarking cohort (20 out of 119 cases). Structural variants are ranked by GEM based on the genes harbored by the variant and presented alongside other ranked genes with coding SNVs or small indels based on the top scored gene. The asterisk indicates genes that in the literature are candidates for the phenotype of the diagnostic disease/syndrome (as described in OMIM). The results show that GEM can analyze both deletions (del) and duplications (dup) of sizes as small as 4 kb and up to entire chromosome arms, diverse modes of inheritance, pedigree structure, and from either WGS or WES assay data. GEM also automatically identified compound heterozygotes between SVs and SNV/indels (cases 1, 2, and 8). Input SV calls can include breakpoint-based calls (here “SV”), or imprecise CNV calls based on read depth analysis. Notably, GEM can also infer SVs directly from the small variant data when external SV calls are not provided (cases 2, 10, 15, and 17), and score them appropriately, identifying diagnostic variants that in the original cases were found by microarrays and not by sequencing

Case no.	Top scored gene(s)	Gene rank	GEM score	Variant(s) position	SV type	Length (kb)	Mode of Inheritance	Pedigree type	Assay type	SV calls in input	Diagnosis
252268	FANCA*	1	2.28	chr16:89847864-89863349; FANCA: c.3788_3790delTCT	Del	15	Recessive	Trio	WGS	SV	Fanconi anemia
223449	TANGO2*	1	2.13	chr22:20028937-20057143; TANGO2: c.605+1G>A	Del	28	Recessive	Trio	WGS	None	MECRCN
266523	BTRC*	1	2.05	chr10:102941001-103430600	Dup	490	Dominant	Duo	WGS	SV	Split hand/foot malformation type 3
267392	HIRA, TBX1*	1	2.05	chr22:18893883-21562619	Del	2669	Dominant	Single	WES	CNV	DiGeorge syndrome; velocardiofacial syndrome
267148	KMT2A	1	1.87	chr11:116691508-126432828; chr22:17038511-20307516	Dup	9741; 3269	Dominant	Trio	WES	CNV	Emanuel syndrome
253691	HIRA, TBX1*	1	1.73	chr22:18893883-20307516	Del	1414	Dominant	Single	WES	CNV	DiGeorge syndrome; velocardiofacial Syndrome
256943	MAGEL2*	1	1.64	chr15:22833478-28566610	Del	5733	Dominant	Single	WES	CNV	Prader Willi syndrome
254012	NDUFS3*	1	1.56	chr11:47605229-47609177; NDUFS3: c.374G>A	Del	4	Recessive	Trio	WGS	SV	Leigh syndrome
254728	EPHA4	2	1.46	chr2:220309089-224580863	Del	4272	Dominant	Single	WGS	SV	Pathogenic deletion in 2q35q36.1
44671	NPAP1	1	1.42	chr15 tetrasomy (broken in multiple dups)	Dup	4542; 991; 358; 158	Dominant	Trio	WGS	None	Isodicentric chromosome 15 syndrome
360547	FREM1	1	1.33	chr9:1-18477200	Del	18,437	Dominant	Trio	WGS	SV	Chromosome 9p deletion syndrome
259685	TYROBP	1	1.31	chr19:23158251-33502767	Dup	10,345	Dominant	Trio	WES	SV	Partial trisomy 19p12.q13.11
266700	TAB2	1	1.31	chr6:144951601-150260400	Del	5309	Dominant	Trio	WGS	SV	Chromsome 6q24-q25 Syndrome
244102	MAGEL2*	1	1.28	chr15:23684685-26108259	Del	2424	Dominant	Single	WES	CNV	Prader Willi syndrome
204560	JAG1*	2	1.21	chr20:10471400-13459333	Del	44	Dominant	Trio	WGS	None	Alagille syndrome
246146	HCN1	1	1.20	chr5:213101-46,270,700	Dup	44	Dominant	Single	WGS	SV	Trisomy 5p
45020	PCDH19*	1	1.15	chrX:92925011-99669272	Del	6744	X-linked dominant	Trio	WGS	None	Developmental and epileptic encephalopathy 9
248678	FANCC*	1	1.14	chr9:97998556-98009092	Del	11	Recessive	Single	WGS	SV	Fanconi Anemia
352726	THRA	1	1.00	chr17:32147833-79020944	Dup	46,873	Dominant	Proband	WGS	SV	Distal trisomy 17q
251355	TRIP11	4	0.58	chr14:84783523-96907490	Del	12,124	Dominant	Duo	WGS	SV	Chromosome 14q31.2q32.2 Syndrome

GEM gene scores are Bayes factors (BF) [84]; these were used to rank gene candidates (Additional file 2: Figure S1). BFs are widely used in AI, as they concisely quantify the degree of support for a conclusion derived from diverse lines of evidence. In keeping with established practices [84], a BF of 0–0.69 was considered moderate support, 0.69–1.0 substantial support, 1.0–2.0 strong support, and above 2.0, decisive support [84]. Scores less than 0 indicated support for the counter hypothesis—that variants in that gene were not causal for the proband’s disease. GEM outputs also include several annotations and metrics that provide additional, supportive guidance for subsequent expert case review (Additional file 2: Figure S1). Experience has shown that such guidance is critical for adoption by experts who wish to review the evidence supporting automated variant assertions. These include VAAST, VVP, and Phevor posterior probabilities, conditioned upon proband sex, gene location, and ancestry. Annotations include variant consequence, ClinVar database pathogenicity assessments, and OMIM conditions associated with genes. This metadata enables expert users to review the major contributions underpinning a final GEM score. Moreover, GEM prioritizes diplotypes, rather than variants, which speeds interpretation of compound heterozygous variants in recessive diseases (Additional file 2: Figure S1B). Comparison of the diagnostic performance of GEM to variant prioritization methods utilized ranking of the correct diagnostic gene. We assumed that in the case of compound heterozygotes, variant prioritization methods such as Exomiser would rank one variant of the pair highly, leading to identification of the other upon manual review (“Methods”).

**Fig. 1:**
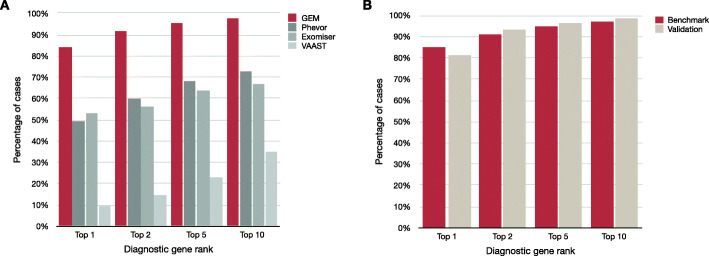
The diagnostic sensitivity of GEM was greater than the variant prioritization methods Phevor, Exomiser, and VAAST. **A** Proportion of the benchmark cohort of 119 cases where the true causal genes (or variants in the case of causal SVs) were identified among the top 1st, 2nd, 5th, or 10th gene candidates. Patient phenotypes were extracted manually from medical records by clinicians and provided as HPO term inputs to GEM, Exomiser, and Phevor. VAAST only considers variant information. It should be noted that GEM and Phevor ranks correspond to genes, which may include one or two variants (the latter in the case of a compound heterozygote), whereas Exomiser and VAAST ranks were for single variants. In the case of compound heterozygotes, the rank of the top-ranking variant is shown for Exomiser and VAAST. **B** Comparison of GEM performance in the validation cohort (excluding SV cases) versus the validation cohort (comprised of 60 rare genetic disease cases from multiple sources)

GEM ranked 97% of previously reported causal gene(s) and variant(s) among the top 10 candidates in the 119 benchmark cohort cases. In 92% of cases, it ranked the correct gene and variant in the top 2 (Fig. 1A). By comparison, the next best algorithm, Phevor, identified 73% of causal variants in the top 10 candidates and 59% in the top 2. GEM, Phevor, and Exomiser prioritize results by patient phenotypes (provided as HPO terms) in addition to variant pathogenicity, whereas VAAST only utilizes genotype data, explaining its lower performance. Thus, these data also highlight that patient phenotypes improve the diagnostic performance of automated interpretation tools.

The benchmark cohort included 3 cases for which two genes were reported to contribute to the patient phenotype. This rate (2.5%) is consistent with previous reports for digenic inheritance [85]. The statistics above use the top ranked genes in these cases, but Additional file 1: Table S3 shows that GEM also ranked the second causal gene among its top candidates, whereas Phevor reported poor ranks in one case, and Exomiser missed the second gene in two out of the three cases.

Next, we investigated whether the diagnostic performance of GEM extended to Mendelian diseases other than those of NICU infants, such as patients with later disease onset, less severe presentations, or with data produced by other variant calling pipelines or outpatient genetic clinics. For these analyses, we compiled a validation cohort largely consisting of WES cases from five different academic medical centers (Table 1; Additional file 1: Table S2). The diagnostic performance of GEM in the validation cohort was almost identical to that in the benchmark cohort (Fig. 1B). These data demonstrated that the diagnostic performance of GEM was not dependent of disease severity, age of onset, or genomic sequencing or variant detection methods.

An implication of these findings is that GEM achieved 97% recall (true positive rate) by review of 10 genes, whereas the other tools had < 78% recall by similar review (Fig. 1, Additional file 2: Figure S2). In part, this difference reflected the unique ability of GEM to prioritize SVs. Excluding SV cases, GEM, Phevor, and Exomiser achieved recall of 97%, 83%, and 76%, respectively, by review of 10 genes (Additional file 2: Figure S3A). Furthermore, VAAST and Exomiser failed to provide rankings for 4 and 18 true positive variants, respectively. Exclusion of false negatives and SV cases increased the top 10 recall of Exomiser to 93% (Additional file 2: Figure S3B), in agreement with previous reports [86]. These data show the importance of including all types of cases and causal variants in benchmarking to avoid overestimation of diagnostic performance in real-world clinical applications.

### Scoring of structural variants increases diagnostic rate

A major barrier to the incorporation of SV calls into genome diagnostic interpretation, whether manual or using eCDSS, is their low precision (high false positive, FP, rates) using short read alignments, with typical FP rates of 20–30% [87, 88]. This leads to overwhelmingly time-consuming, manual assessment of event quality and significance for large numbers of SVs. GEM minimizes the effect of low precision by scoring SVs either with SV calls provided in the proband’s input VCF file, and/or by inferring ab initio their existence from metadata associated with SNV and indel calls (“Methods”; see below). The benchmark cohort included 20 cases in which SVs were reported to be causative, reflecting a similar incidence to that in real-world experience (Fig. 1A, Table 2) [20–23]. In 17 of these, the causative SV was ranked first by GEM. In two, it was ranked second, and in one it was listed fourth, demonstrating that GEM retains adequate diagnostic performance with imprecise SV calls. The disease-causing SVs in the benchmark set ranged from small (4 kb) to very large (e.g., entire chromosome arms). In three cases, the diagnosis was of an autosomal recessive disorder in which the SV was compound heterozygous with a SNV/indel. In each, GEM integrated the two variants correctly, automatically identifying the causative diplotypes (Additional file 2: Figure S5). With regard to the diagnostic specificity of GEM, the mean and median number of gene candidates for these probands with BF > 0 (any support) was 8.7 and 9.5, respectively, which was similar to probands whose VCF files contained no SVs, causative or otherwise.

Large SVs frequently affect more than one gene. For consistency with other variant classes, genes within multigenic SVs are grouped and sorted by GEM based upon the gene-centric Bayes factor score associated with the overlap of the proband phenotype and known Mendelian disorders (if any) associated with them (“Methods”). For example, Additional file 2: Figure S4 shows a case that highlights the practical utility of prioritizing genes harboring causative SVs together with SNVs and short indels in the same report, rather than separately cross-referencing with databases of microdeletion syndromes [89]. While it is often unknown which genes harbored in a pathogenic SV are causal for microdeletion/microduplication syndromes, GEM’s gene-by-gene rankings typically agreed with causal gene candidates suggested by the literature (asterisks in Table 2).

By default, GEM evaluates every gene and transcript for the presence of overlapping SVs. Notably, four benchmark cases did not include externally called SVs in their input VCFs (these had been previously diagnosed by manual inspection and orthogonal confirmatory tests; Table 2). Nevertheless, GEM inferred the existence of these four SVs using its ab initio SV identification algorithm and evaluated them jointly with SNVs and indels (“Methods”). To further demonstrate this innovative functionality, we removed all external SV calls from each input VCF file of the 14 WGS cases (as GEMs ab initio SV imputation is currently limited to WGS data) and reran GEM. GEM re-identified 13 of the 14 of the causative SVs. Although GEM’s inferred SV termini were imprecise, an overlapping SV of the same class (duplication, deletion, or CNV) and ploidy to that in the original VCF was inferred, and the same high scoring gene and mode of inheritance/genotype (autosomal dominant, simple recessive, or compound heterozygote) was ranked first. SV recall within the top 1, 5, and 10 ranked GEM results were 71%, 86%, and 93%, respectively. The single false negative was a small (4 kb) homozygous deletion. GEM failed to identify this SV because it did not span sites with known variation in the gnomAD database [73], upon which ab initio SV inference is based (“Methods”). With regard to specificity, the mean and median number of results with genes with BF > 0 in these cases was 10.6 and 12.5, respectively. These values differed only slightly from the results obtained using external SV calls (8.7 and 9.5, respectively), despite the fact every gene and transcript was evaluated for the presence of SVs.

Collectively, these results demonstrate the accuracy of GEM’s ab initio approach to identification and prioritization of SVs without recourse to external calls and databases of known causative SVs. Thus, GEM compensates, in part, for the low recall of SVs from short-read sequences. If an external SV calling pipeline fails to detect an SV, there is still the possibility that GEM will identify it via this ab initio approach. This capability, together with GEM’s ability to accurately prioritize SVs in the context of SNVs and short indels, addresses an unmet need for clinical applications. This characteristic also makes GEM well suited for reanalyses of older cases and/or pipelines lacking SV calling.

### Leveraging automated phenotyping from clinical natural language processing

Ontology-based phenotype descriptions, using Human Phenotype Ontology (HPO) terms [69], are widely used to communicate the observed clinical features of disease in a machine-readable format. These lists of terms are usually derived by manual review of patient EHR data by trained personnel, a time-consuming, subjective process. A solution is automatic extraction of patient phenotypes from clinical notes using clinical natural language processing (CNLP) [28, 90]. One challenge has been that CNLP generates many more terms than manual extraction. Thus, manual curation yielded an average of 4 HPO terms (min = 1, max = 12) in the benchmark cohort, while CNLP yielded an average of 177 HPO terms (min = 2, max = 684). Some of the extra CNLP terms are hierarchical parent terms of those observed, raising the concern that their inclusion diminishes the average information content in a manner that could impede diagnosis [27]. To investigate the effect of CNLP-derived HPO terms on GEM’s performance, we analyzed the benchmark cohort both with HPO terms extracted by commercial CNLP (“Methods”) and manually extracted HPO terms.

**Fig. 2:**
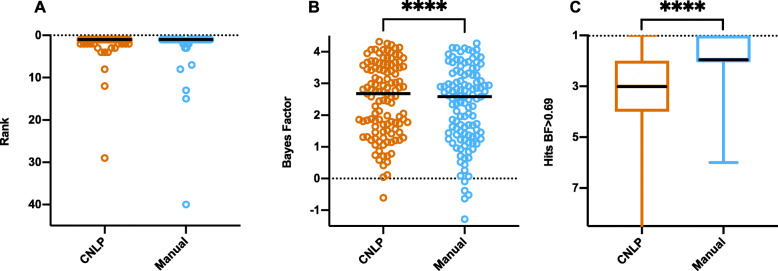
Comparison of GEM performance with manually curated and CNLP-derived HPO terms in the benchmark cohort. Distribution of ranks for causal genes (**A**); GEM Bayes factors for causal genes (**B**); and number of candidates (hits) at BF ≥ 0.69 threshold (moderate support) (**C**). The black line in the graphs denotes the median. The asterisks represent statistical difference between the groups with *p* < 0.0001 from a two-tailed Wilcoxson matched pairs signed rank test (ranks showed no statistically significant difference)

Figure 2 shows the distributions and medians for ranks and GEM gene scores of true positives, as well as the number of gene candidates with BF ≥ 0.69 (moderate support), for manual and CNLP terms. The median rank of the causal genes did not significantly differ between CNLP- and manually derived phenotype descriptions (Fig. 2A). The median GEM gene score of true positives was higher with CNLP-derived phenotypes than with manual phenotypes (Fig. 2B). The number of candidates above the BF threshold was higher with manual phenotypes than CNLP (Fig. 2C). CNLP rescued a few true positives with low ranks and negative BF scores compared to manual phenotype descriptions (Fig. 2A, B). These results demonstrate that GEM performs somewhat better with CNLP-derived phenotype descriptions as part of an automated interpretation workflow, than with sparse, manual phenotypes.

### Resilience to mis-phenotyping and gaps in clinical knowledge

**Fig. 3:**
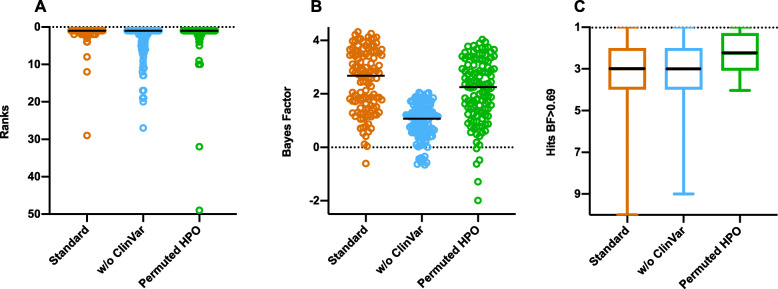
Impact of missing data and mis-phenotyping on GEM performance in the benchmark cohort. Causal gene rank (**A**); Bayes factors for causal genes (**B**); and number of candidates (hits) above gene BF ≥ 0.69 threshold (moderate support) (**C**) under standard conditions, withdrawing ClinVar information, and permuting HPO terms extracted by CNLP. The black line in the graphs denotes the median

Given the potentially noisy nature of the CNLP phenotype descriptions, it was important to examine the sensitivity of GEM to mis-phenotyping. To address this question, we randomly permuted CNLP-extracted HPO terms between cases, weighting by term frequency within the cohort, so that every case maintained the same number of HPO terms as CNLP originally provided. Permuting HPO terms resulted in lower gene scores, and several cases would have been lost for review had the gene score threshold of BF ≥ 0 still been used, but ranks are unaffected (98% in top 10; Fig. 3). This represented lower bound estimates, as actual mis-phenotyping (short of data tracking issues) would be much less. It is also worth noting that even using randomly permuted phenotype descriptions, GEM’s performance still exceeded that of Phevor and Exomiser using the correct phenotypes (Additional file 2: Figure S2). We therefore conclude that GEM is resilient to mis-phenotyping.

We also evaluated the impact of gaps in clinical knowledge on GEM performance by withdrawing annotations from a key clinical database, ClinVar. Absence of ClinVar annotations had minimal impact in ranking, although it reduced median gene scores (1.1 vs. 2.7), resulting in 9 cases no longer meeting the minimum Bayes factor threshold ≥ 0 (any support; Fig. 3). Clearly, ClinVar provided GEM with valuable information. Nonetheless, without ClinVar, GEM’s top 10 maximal recall (88%) still exceeded that of Phevor (72%) and Exomiser (65%; Fig. 1). More broadly, these results show that integrating more datatypes in GEM improves diagnostic performance and results in greater algorithmic stability (Figs. 2 and 3).

About 70% (86/122) of the disease-causing variants in the benchmarking dataset are reported in ClinVar with pathogenic (P) or likely pathogenic (LP) clinical significance annotations. Moreover, each proband’s whole-genome variant set contained on average 1.9 variants with ClinVar P/LP annotations. These two facts underscore the importance of ClinVar annotations for assisting diagnosis. They also make clear that tools that leverage ClinVar information need to avoid false positives which lead to longer candidate lists as non-causal genes also contain ClinVar P/LP variants. Additional file 1: Table S4 breaks down results for the benchmark cohort with respect to ClinVar annotations of causal variants. Overall, mean, and median ranks were slightly improved for diagnostic variants with ClinVar P/LP annotations *vs*. those without them (mean 1 *vs.* 3), with GEM showing the greatest improvement in ranks. Moreover, GEM maintained the same number of candidates with GEM gene score > 0 for both classes [10], demonstrating that GEM can use ClinVar status to improve diagnostic rates without increasing the number of candidates for review.

### GEM performs equivalently on parent-offspring trios and single probands

Parent-offspring trios are widely used for molecular diagnosis of rare genetic disease. While a recent study showed that singleton proband sequencing returned a similar diagnostic yield as trios [91], interpretation of trio sequences is less labor-intensive. For example, trios enable facile identification of de novo variants, which is the leading mechanism of genetic disease in outbred populations [92]. Likewise, in recessive disorders, proband compound heterozygosity can be automatically distinguished from two variants in *cis*. However, these benefits are associated with increased sequencing costs. Moreover, both parents are not always available for sequencing or do not wish to have their genomes sequenced.

**Fig. 4:**
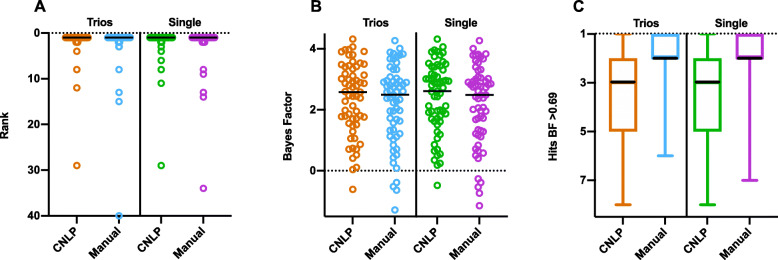
Comparative performance of parent-offspring trios or duos vs. singleton probands in the benchmark cohort. Causal gene rank (**A**); Bayes factors (**B**); and number of candidates (hits) above gene BF ≥ 0.69 (moderate support) (**C**) for 63 cases analyzed as parent-offspring trios (*n* = 59) or duos (*n* = 4), as compared with analysis as single probands, using both manually curated or CNLP-derived HPO terms. The black line in the graphs denotes the median. No statistically significance difference between the any manual/CNLP groups was found between trios versus single probands using the two-tailed Wilcoxson matched pairs signed rank test

To understand how GEM performs in the absence of parental data, we reanalyzed the 63 trio and duo cases from the benchmark cohort as singleton proband cases. Surprisingly, we observed insignificant differences in the mean rank of the causal gene (Fig. 4A), GEM score of the causal gene (Fig. 4B), or number of candidates with BF ≥ 0.69 (Fig. 4C), using either manually or CNLP-extracted HPO terms. In contrast, this reanalysis was associated with a decline in the performance of Exomiser (Additional file 2: Figure S6). These analyses demonstrated that GEM was resilient to the absence of parental genotypes, a feature that could increase the cost effectiveness and adoption of WGS.

### GEM scores optimize case review workflows

Conventional prioritization algorithms rank variants, enabling manual reviewers to start with the top ranked variants, and work their way down in the list until a convincing variant is identified for further curation, classification, and possible clinical reporting. This review process typically involves (a) assessing variant quality, deleteriousness, and prior clinical annotations; (b) evaluating whether there is a reasonable match between the phenotypes exhibited by the patient and those reported for condition(s) known to be associated with defects in the corresponding gene; and (c) considering the match in mode(s) of inheritance reported in the literature for the candidate disease and the patient’s diplotype.

GEM accelerates this process, because it intrinsically considers variant quality, deleteriousness, prior clinical annotations, and mode of inheritance. Furthermore, at manual review, GEM gene scores summarize the relative strength of evidence supporting the hypothesis that the gene is damaged and that this explains the proband’s phenotype.

GEM scores provide a logical framework for setting thresholds with regard to the optimal number of candidates that should be reviewed to achieve a desired diagnostic rate. This enables laboratory directors and clinicians to dynamically set optimal tradeoffs of interpretation time and diagnosis rate for specific patients, relative to their suspicion of a genetic etiology or results of other diagnostic tests.

**Fig. 5:**
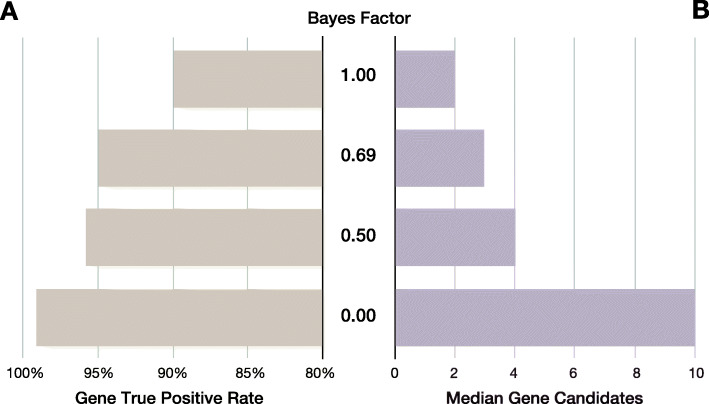
Trade-off between GEM gene scores, maximal true positive rates, and number of candidates for review in the benchmark cohort. GEM gene scores are Bayes factors (BF) that can be used speed case review. **A** Gene maximal true positive rate achieved at the different BF thresholds (*Y*-axis). **B** Median number of candidate genes for review at each BF threshold. As the BF threshold is decreased, true positive rate increases, while the number of candidates to review remains manageable. Input HPO terms for this analysis were extracted by CNLP

We examined the effect of different BF thresholds on recall (true positive rate) and median number of gene candidates for review in the benchmark cohort (Fig. 5). In such analyses, it is germane to consider the concept of *maximal true positive rate (or recall)* to measure the theoretical proportion of true positive diagnoses recoverable by perfect interpretation when reviewing a set of *N* genes containing the true positive. For example, in the benchmark dataset, a GEM causal gene score threshold ≥ 0 would retain a median of ten candidates for review and provide a 99% maximal recall; whereas a threshold of ≥ 0.5 would retain a median of four candidates for review for a 97% maximal recall (Fig. 5).

These results illustrate how a tiered approach to case review using GEM gene scores could minimize the number of candidate genes to review, and, thereby manual interpretation effort. For example, a first pass review of candidates with a gene BF ≥ 0.69 provided an expected 95% diagnostic rate (and a corresponding median of 3 genes to be manually reviewed). If followed by a second pass using a threshold > 0, if no convincing candidates are found, an additional 4% possible diagnoses would be recovered, involving review of a median increment of seven genes. Application of this two-tiered approach to the benchmark dataset of 119 cases (Fig. 1), required manual final review of 395 candidate genes (3 genes in 115 cases and 10 genes in 5 cases), or an average of 3.3 candidate genes per case, for a maximal recall of 99%. Finally, review of candidates with BF < 0 recovered the last true positive in the benchmark cohort (*COL4A4*, ranked 40th in the GEM report with a BF = − 0.6. This case was a phenotypically and genotypically atypical autosomal dominant presentation of Alport syndrome 2 (MIM 203780).

### Clinical decision support for diagnosis

Quantifying how well the observed phenotypes in a patient match the expected phenotypes of Mendelian conditions associated with a candidate gene is challenging for clinical reviewers and is a major interpretation bottleneck. In practice, clinicians look for patterns of phenotypes, biasing their observations. In addition, patient phenotypes evolve as their disease progresses. And there is considerable, disease-specific heterogeneity in the range of expected phenotypes. Simply comparing exact matches of the patient’s observed HPO terms with those expected for that disease is suboptimal, because the observed and expected HPO terms are often hierarchical neighbors, rather than exact matches. Missing terms, particularly those considered pathognomonic for a condition, and “contradictory” terms further complicate such comparisons by clinicians. Thus, generation of quantitative, standardized, unbiased models of disease similarity has proven elusive.

GEM can automate or provide clinical decision support for this process via a condition match (CM) score (“Methods”). The GEM CM score summarizes the match between observed and expected HPO phenotypes for genetic diseases and considers the known mode(s) of inheritance, associated gene(s), their genome location(s), proband sex, the pathogenicity of observed diplotypes, and ClinVar annotations. Importantly, CM scores reflect relationships between phenotype terms as expressed in the HPO ontology graph, enabling inclusion of imprecise matches in similarity comparisons. CM scores can be used in a wide variety of clinical settings to prioritize and quickly assess possible Mendelian conditions as candidate diagnoses, a process we term *diagnostic nomination*.

Specific, definitive, genetic disease diagnosis remains a significant challenge for clinical reviewers, even with the short, highly informative candidate gene lists provided by tools such as GEM. This is because many genes are associated with more than one Mendelian disease. For example, application of a GEM causal gene score threshold *≥* 0.69 to the 119 probands in the benchmark cohort results in a median of 3 gene candidates per proband (c.f. Fig. 5), associated with a maximal gene recall of 95%. However, because many genes are associated with more than one disease, clinical reviewers would actually need to consider around 12 candidate Mendelian conditions per proband (data not shown). This difficulty is exacerbated by the fact that most laboratory directors are not physicians and lack formal training in clinical diagnosis.

**Fig. 6:**
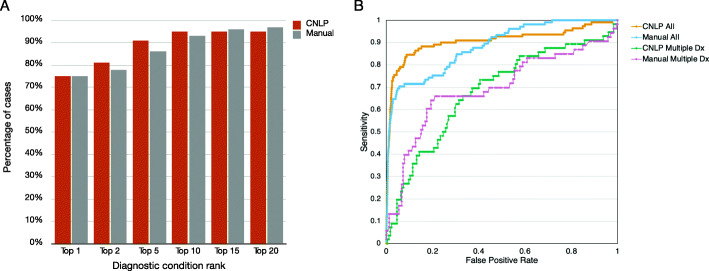
Performance of GEM condition match scores for diagnostic nomination in the benchmark cohort. **A** Ranks for reported diagnostic conditions for the benchmark dataset, using a GEM gene BF score ≥ 0.69 and sorted by CM score, for HPO terms derived from CNLP or manual curation. **B** Receiver-operator characteristic curves for the condition match **(**CM) score for all hits with BF *≥* 0. CNLP All: HPO extracted from clinical notes by CNLP; AUC = 0.91. Manual: Manually curated HPO terms; AUC = 0.88. CNLP Multiple Dx: CNLP-derived CM score for the true positive disorder versus the other possible disorders associated with that gene; AUC = 0.68. Manual Multiple Dx: As for CNLP-derived CM but using manually curated HPO terms; AUC = 0.69

Determination of a specific, definitive genetic disease diagnosis among several candidates can be accomplished with a combination of GEM CM scores and causal gene scores (Fig. 6). Using the benchmark cohort’s true (reported) gene and disorder diagnoses as ground truth, we used a GEM gene score threshold ≥ 0.69 to recover gene candidates, and the associated CM scores to rank order the diseases associated with those gene candidates (Fig. 6A). Using CNLP-derived phenotypes, the true disease diagnosis was the top nomination by CM score in 75% of cases, within the top 5 in 91% of cases, and within the top 10 in 95% of cases. Performance was inferior with manually extracted phenotype terms. The area under the receiver-operator characteristic (ROC) curves (AUCs) were 0.90 and 0.88, for CNLP and manual terms, respectively (Fig. 6B). This implied that the larger number of CNLP-extracted terms conveyed greater information content, permitting better discrimination of the correct diagnostic condition, than sparse, manually extracted phenotypes [27].

In the benchmark cohort, 58 of the 100 candidate genes (excluding cases with causal, multigenic SVs) were associated with 2 or more disorders (median of 3 gene-disorder, maximum of 15; Additional file 2: Figure S7 shows the example of *ERCC6*). We measured how well the CM score distinguished between multiple, alternative disorders associated with the same gene (Fig. 6B). In these 58 cases, the AUC was less than that for CNLP with the entire set of candidate genes in the benchmark cohort (0.68 vs 0.9). This decrease can be at least partially explained by the high similarity (and in some cases identity) of the clinical features of different disorders associated with the same gene. Thus, a combination of GEM gene and CM scores can refine candidate disorders for clinical reporting, further reducing review times.

### Reanalysis of previously unsolved cases

Recent reports show that reanalysis of older unsolved cases suspected of rare genetic disease can yield new diagnoses supported by incremental increases in knowledge of pathogenic variants, disease-gene discoveries, and reports of phenotype expansion for known disorders [93, 94]. While worthwhile, there are barriers to reanalysis, such as limited reimbursement and low incremental diagnostic yield, that limit use to physician requests. Ideally, all unsolved cases would be reanalyzed automatically periodically, and a subset with high likelihood of new findings would be prioritized for manual review. The strong correlation between true positive rates and GEM gene scores (Fig. 5) suggested a strategy for triaging reanalyzed cases for manual review: only cases for which the recalculated GEM score had increased sufficiently to suggest a high probability of a new diagnosis would pass the threshold for manual review. Likewise, GEM condition match scores could be used to search all prior cases to identify the subset of unsolved cases with support for particular Mendelian conditions, aiding cohort assembly for targeted reanalysis based upon particular proband phenotypes, or for review by particular medical specialists. Of note, an advantage of CNLP is that it is possible to automatically generate a new clinical feature list at time of reanalysis. This is particularly important in disorders whose clinical features evolve with time and were the observed features may be nondescript at presentation.

To test the utility of GEM for reanalysis, we selected 14 unsolved cases that had rWGS performed by RCIGM. For these reanalyzes, we used CNLP-derived HPO terms (Table 3) and a more stringent gene BF threshold ≥ 1.5 to restrict the search to very strongly supported candidates. Ten cases yielded no hits. Four cases returned a total of 7 candidate genes. Review of three cases did not return new diagnoses. In the remaining case, a new likely diagnosis was made of autosomal dominant Shwachman-Diamond Syndrome (MIM: 260400) or severe congenital neutropenia (MIM: 618752) [95, 96], both of which are associated with pathogenic variants in *SRP54*. The respective CM scores using 261 CNLP-derived terms were relatively high (0.893 and 0.672, respectively). The association of *SRP54* and these disorders was first reported in November 2017 [95] and entered in OMIM in January 2020 [97], which explained why it was not identified as the diagnosis originally in July 2017. The identified candidate p.Gly108Glu variant has been classified as “uncertain significance” by ACMG guidelines. However, if we were able to confirm de novo origin with paternal genotypes (which is currently lacking for this single proband case), the variant could be reclassified as “likely pathogenic” (meeting PM2, PM1, PP3, and PM6 of the ACMG guidelines). This was a singleton proband sequence and confirmation is being pursued. Thus, GEM reanalysis of 14 unsolved cases led to 7 gene-disorder reviews (an average of 0.5 per case), and yielded one likely new diagnosis, which was consistent with prior reanalysis yields [93, 94].

**Table 3:** Previously undiagnosed cases with potential leads. Cases with hits with a GEM gene score BF > 1.5. *Zygo* zygosity, *Hom* homozygous, *Het* heterozygous, *Dup* large duplication

Case	Pedigree	Sex	Assay	Rank	Chr	Gene	Variant Type	Variant ACMG	De novo	Zygo	GEM score	Mode of inheritance	MIM ID(s)	CM score(s)
244799	Single	Male	WGS	1	14	SRP54	SNV	Uncertain significance	Likely	Het	1.76	Dominant	618752, 260400	0.672, 0.893
245237	Trio	Male	WGS	2	X	GK	SNV	VUS	Yes	Het	1.60	X-linked recessive	307030	1.119
245237	Trio	Male	WGS	3	16	FANCA	SNV	VUS	No	Hom	1.55	Recessive	227650	1.315
245768	Single	Male	WGS	1	16	TSC2	Dup	VUS	Likely	Het	1.64	Dominant	N/A	N/A
247458	Single	Male	WGS	1	1	SLC25A24	SNV	VUS	Likely	Het	1.86	Dominant	612289	1.995
247963	Trio	Female	WGS	1	X	STAG2	SNV	Likely pathogenic	Yes	Het	1.53	X-linked dominant	301022	1.25

## Conclusions

Here we described and benchmarked a Bayesian, AI-based gene prioritization tool for scalable diagnosis of rare genetic diseases by CNLP and WES or WGS. GEM improved upon prior, similar tools [19, 27, 28, 98, 99] by incorporating OMIM, HPO, and ClinVar knowledge explicitly, automatically controlling for confounding factors, such as sex and ancestry, compatibility with CNLP-derived phenotypes, SVs and singleton probands, and by directly nominating diplotypes and disorders, rather than just prioritizing variants.

In the cohorts examined, GEM had maximal recall of 99%, requiring review of an average of 3 candidate genes, and less than one half of the associated disorders nominated by other widely used variant prioritization methods per case. Improved diagnostic performance is anticipated to enable faster and more cost-effective, tiered reviews. GEM recall was essentially unaltered in the absence of parental genotypes in our data, meaning that full trio-sequencing is not always a requirement for high diagnostic yield. However, our cohort includes only definitively solved cases with 70% of variants already classified as P/LP in ClinVar; identification of less certain candidate variants and genes may still benefit from parental genotypes for ascertaining de novo variants, and for phasing alleles in genes associated to recessive conditions.

Uniquely, GEM provided AI-based unified gene prioritization for SVs and small variants. Hitherto, this has been frustrated by the high false positive rates of SV calls using short-read sequences and lack of a suitable framework for AI-based SV pathogenicity assertions [87, 88]. Furthermore, GEM inferred SV calls ab initio from WGS when they were not provided. These functionalities are critical for reanalyzing older cases, and for pipelines lacking SV calls.

Finally, in a small data set, we showed that GEM can efficiently reanalyze cases, potentially permitting cost-effective, scalable reanalysis of previously unsolved cases as disease, gene, and variant knowledge evolves [94, 100]. Indeed, integration of GEM and CNLP could enable automatic surveillance for rare disease patients [101] from genomes obtained for research or other clinical tests performed in healthcare [102, 103]. These combined features hold promise for reduced time-to-diagnosis and greater scalability for critical applications, such as in seriously ill children in the NICU/PICU [27, 104].

## Supplementary Information

**Additional file 1.** Supplementary Tables (Tables S1-S14).

**Additional file 2.** Supplementary Figures (Figures S1-S7).

## Data Availability

The datasets supporting the conclusions of this article are included within the article and its additional files. Due to patient privacy, data sharing consent, and HIPAA regulations, our raw data cannot be submitted to publicly available databases. Anonymized outputs from GEM [70], Phevor [15], VAAST [14], and Exomiser [16] for the benchmark dataset cases are tabulated in Additional file 1: Tables S5-S8, and GEM for the validation dataset cases in Additional file 1: Table S10. Condition match scores for hits with gene BF > 0 used for Fig. 6 are tabulated in Additional file 1: Tables S11-S14. GEM, Phevor, and VAAST software implementations for versions used in this analysis are part of the Fabric Enterprise analysis platform and are commercially available [70]. Exomiser source code (version 12.1.0) is available on GitHub [105].

## Abbreviations

AI: Artificial intelligence
BF: Bayes factor
eCDS: Electronic Clinical Support System
CNLP: Clinical Natural Language Processing
CM: Condition match score
GEM: Fabric GEM
HPO: Human Phenotype Ontology
OMIM: Online Mendelian Inheritance in Man
SNV: Single-nucleotide variant
Indel: Insertion-deletion
SV: Structural variants
VAAST: Variant Annotation, Analysis and Search Tool
VVP: VAAST Variant Prioritizer
WES: Whole-exome sequencing
WGS: Whole-genome sequencing
