# Supplementary material for: Artificial intelligence enables comprehensive genome interpretation and nomination of candidate diagnoses for rare genetic diseases
Source: Genome Med. 2021 Oct 14;13:153. doi: 10.1186/s13073-021-00965-0 (PMC8515723; doi:10.1186/s13073-021-00965-0)
Supplement: Supplementary file 2 — Additional file 2. Supplementary Figures (Figures S1-S7). [file 13073_2021_965_MOESM2_ESM.pdf]

# Artificial intelligence enables comprehensive genome interpretation and nomination of candidate diagnoses for rare genetic diseases

Francisco M. De La Vega, Shimul Chowdhury, Barry Moore, Erwin Frise, Jeanette McCarthy, Edgar Javier Hernandez, Terence Wong, Kiely James, Lucia Guidugli, Pankaj B. Agrawal, Casie A Genetti, Catherine A. Brownstein, Alan H. Beggs, Britt-Sabina Löscher, Andre Franke, Braden Boone, Shawn E. Levy, Katrin Õunap, Sander Pajusalu, Matt Huentelman, Keri Ramsey, Marcus Naymik, Vinodh Narayanan, Narayanan Veeraraghavan, Paul Billings, Martin G. Reese, Mark Yandell, and Stephen F. Kingsmore.

**Supplementary Figures S1-S7**

Figure S1. Example of GEM output.

Figure S2. Cumulative ranking of benchmark cases by GEM, Phevor, and Exomiser.

Figure S3. GEM AI-CDS outperforms variant prioritization approaches.

Figure S4. GEM report with scored SV example.

Figure S5. Example of compound heterozygote genotype combining an SV with an SNV.

Figure S6. Impact of removing parent information in ranking.

Figure S7. Diagnostic nomination with condition match scores in GEM report.

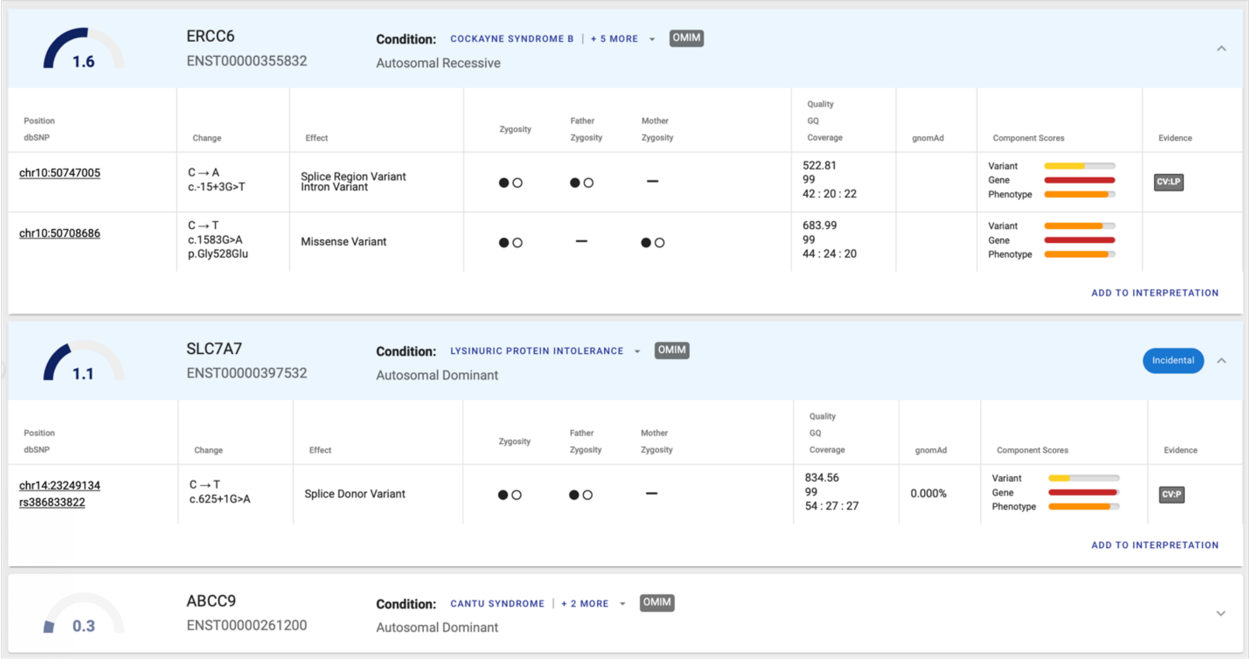


**Figure S1. Example of GEM output.** Each card represents a gene scored by GEM, sorted by the Bayes Factor gene score (large number top left and ideogram), diplotypes are automatically grouped together in a card, and their composite score is shown in the upper left-hand corner of the card. GEM outputs also include several key parameters that provide additional guidance for case review, improving explainability and speeding interpretation, including: gene symbol; canonical transcript ID; mode of inheritance selected by GEM; disease conditions associated with gene (drop-down menu); position of variant; sequence change and HGVS notation; effect of variants in transcript; genotype zygosity in proband and parents (if available); variant quality (QUAL), GQ, coverage, and allele counts; gnomAD allele frequencies (if available); major components scores feeding into GEM: a) 'Variant', variant deleteriousness score (VVP), b) 'Gene', gene burden score (renormalized VAAST score), and c) 'Phenotype', gene phenotype prior (renormalized Phevor gene priors) – all scaled 0-1 and presented logarithmically as color coded bar for ease of presentation; and 'Evidence', ClinVar pathogenic/likely pathogenic evidence, if available. A badge labels a card "incidental," if, although score is high, mode of inheritance may not match what is known about the associated conditions, i.e. the individual is a carrier of a known pathogenic variant.

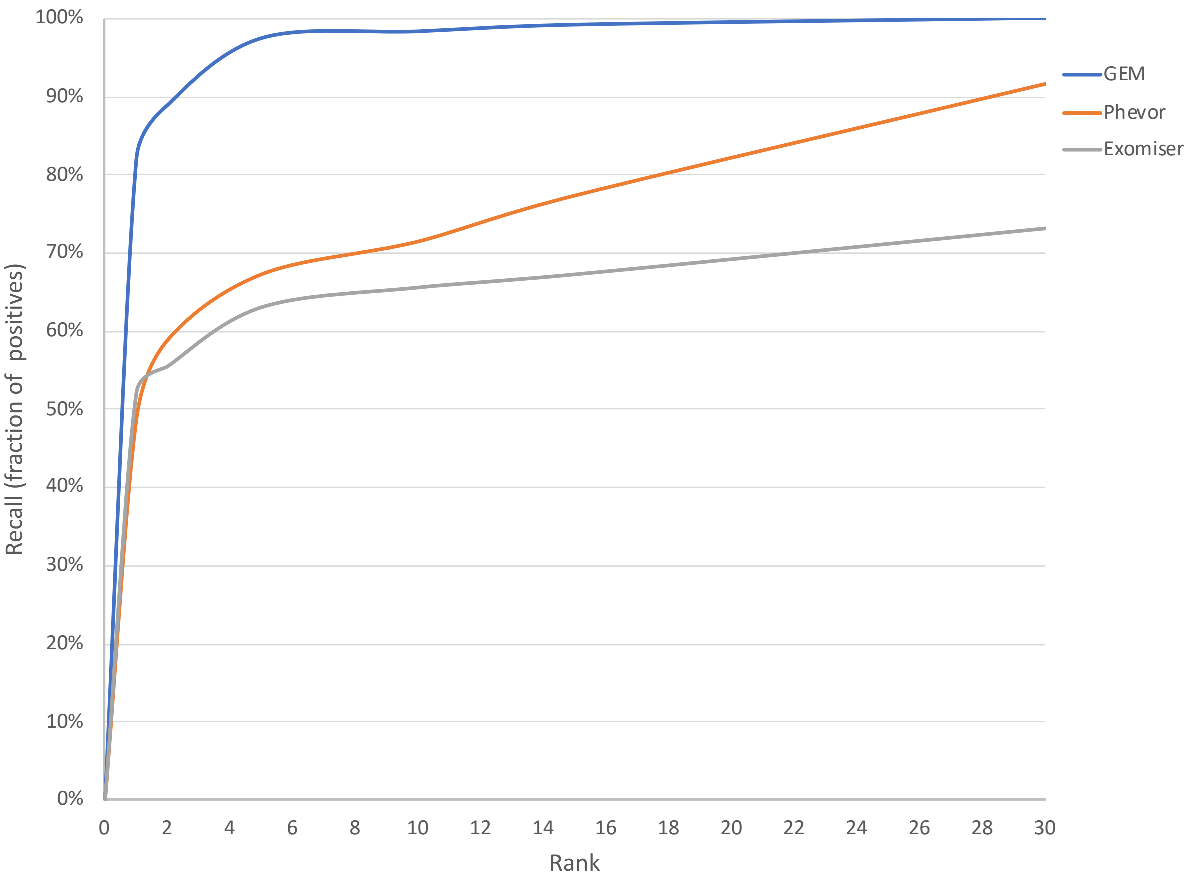


**Figure S2. Cumulative ranking of benchmark cases by GEM, Phevor, and Exomiser.** Percentage of true causal gene (or variant in the case of causal SVs) identified across each rank in the output list the entire benchmark cohort of 119 cases of the benchmark cohort. Patient phenotypes curated by clinicians manually from medical records expressed as HPO terms where provided as inputs to GEM, Exomiser and Phevor. It should be noted that GEM ranks correspond to genes, which may include one or two variants (the latter in the case of a compound heterozygote), whereas the ranks for the other methods are for variants; in the case of compound heterozygotes we indicate the rank of the top-ranking variant for these methods. Ranks were truncated at 30, although Phevor's last rank was 356 whereas Exomiser was 112. Note that Exomiser did not provide true positives in its output for 18 cases and VAAST for 4 cases.

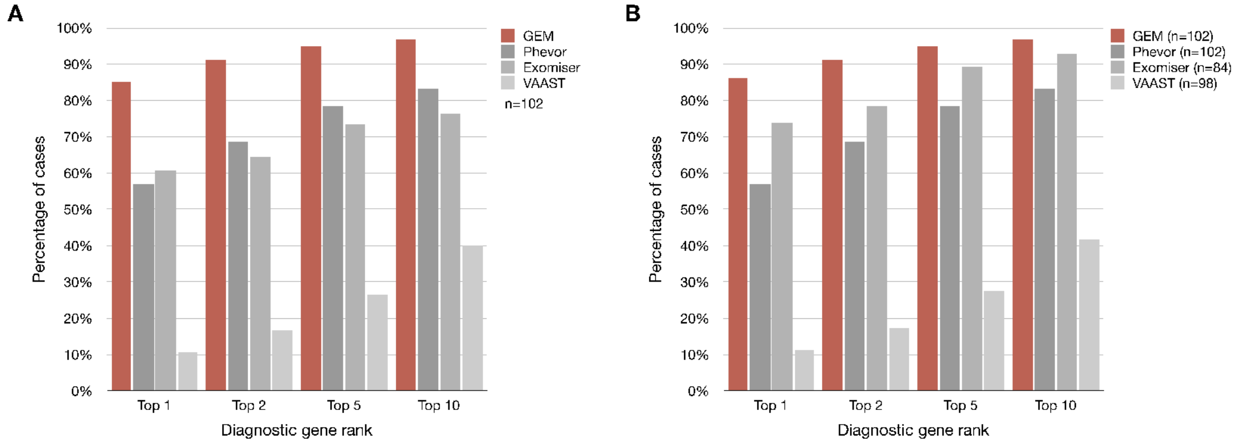


**Figure S3. GEM AI-CDS outperforms variant prioritization approaches.** Comparative performance of GEM's ability to rank diagnostic genes versus those derived from previous variant prioritization algorithms. (A) Percentage of the 102 cases from the benchmark cohort, where SVs were not causal, is identified within the top 1st, 2nd, 5th, or 10th genes by the different methods compared: GEM, Phevor, Exomiser, and VAAST. For the former three methods, patient phenotypes curated by clinicians manually from medical records expressed as HPO terms where provided as inputs; VAAST only considers variant information. It should be noted that GEM ranks correspond to genes, which may include one or two variants (the latter in the case of a compound heterozygote), whereas the ranks for the other methods are for variants; in the case of compound heterozygotes we indicate the rank of the top ranking variant for these methods. (B) Since Exomiser and VAAST were unable to rank the causal variant in a number of cases, here the comparison uses as denominator only the cases each method was able to provide a rank for a causal gene/variant; this should be considered the best possible scenario for each method.

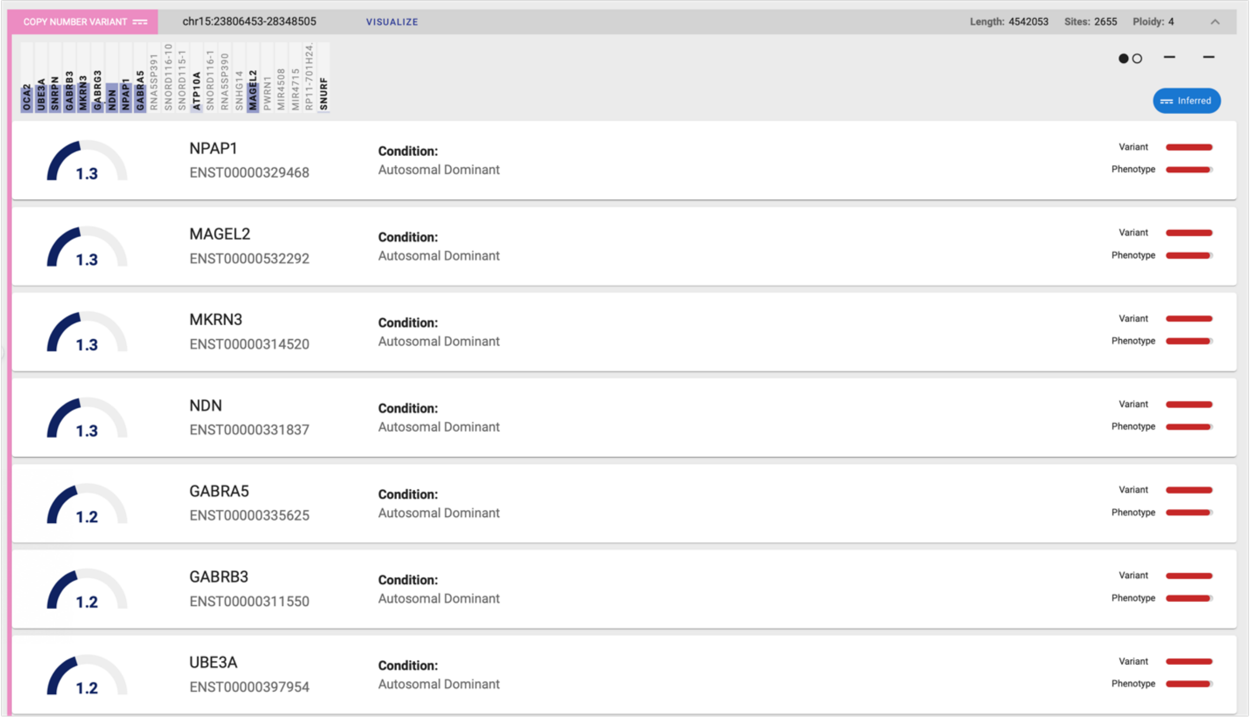


**Figure S4. GEM report with scored SV example.** GEM cards can also represent structural variants and contain overlapped genes scored by GEM and meeting a minimum bayes factor score. Genes within the SV are ranked, and the card can be expanded to visualize an individual gene harbored (as shown) and is listed in the rank of the gene with the highest bayes factor score ("anchor" gene). GEM specifies the type of event (deletion, duplication, copy number change for ploidy greater than 3; genomic coordinates; an ideogram showing the genes overlapped by the event and those genes that were scored by GEM (the height of the bar a succinct representation of the gene Bayes Factor); the length of the event in base pairs; number of SNVs with gnomAD allele frequency data supporting the event (whether the variants were provided on the input VCF or internally inferred ab initio by GEM, like in this case); and estimated ploidy from the data. Component scores shown include a variant deleteriousness score, and the Phevor gene prior, but not burden score, as VAAST cannot presently calculate burden for SVs. The card also indicates if the SV was internally inferred ("Inferred" badge above right). If GEM scores an SV in compound heterozygote state with an SNV or small indel, then the gene card will show details of the small variant as usual. Links are provided for further visualization of the event in the context of annotations relevant to structural variation interpretation such as Decipher Syndromes and ISCA microdeletion/duplication database.

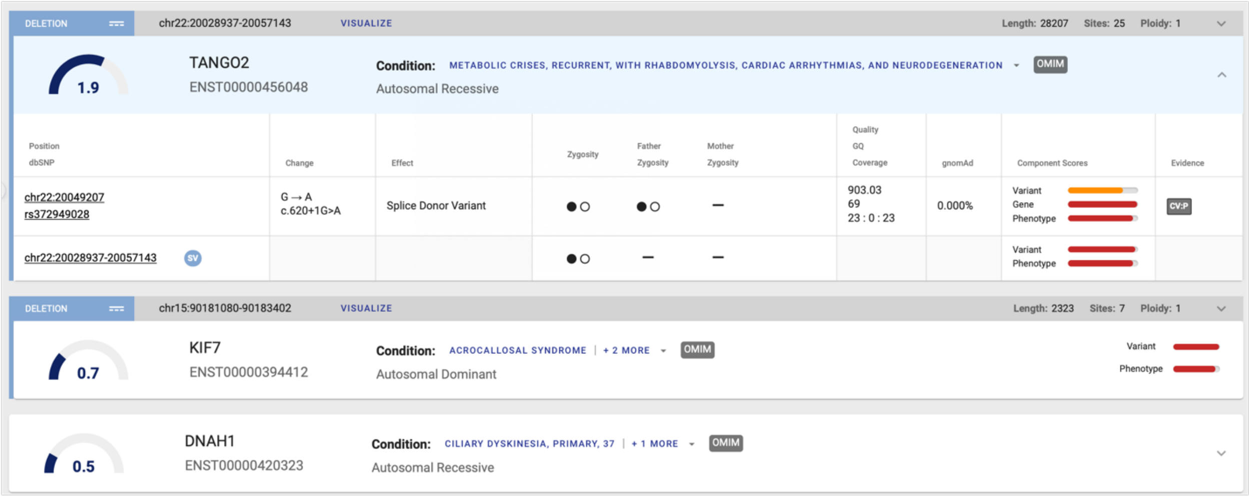


**Figure S5. Example of compound heterozygote genotype combining an SV with an SNV.** *Ab initio* inferred deletion forms a compound heterozygote with an SNV overlapping the gene TANGO2. In this case the SNV shows the usual attributes, whereas the SV shows the interval of the event, its zygosity and its variant and phenotype impact scores. The GEM gene score is calculated for the compound heterozygote recessive genotype.

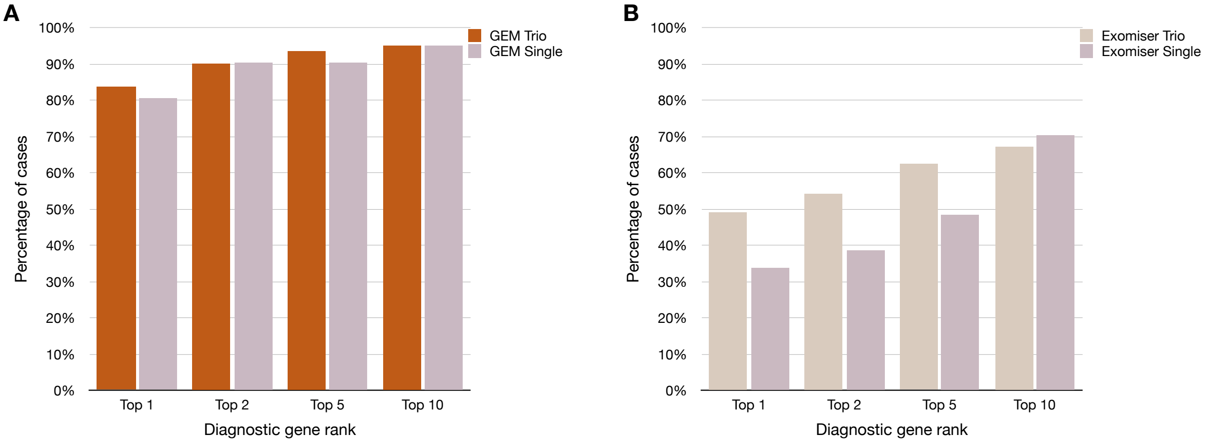


**Figure S6. Impact of removing parent information in ranking.** GEM (Panel A) and Exomiser (Panel B). Exomiser ranking performance drops in the absence of parental genotypes, whereas for GEM has little impact. This analysis is restricted to 63 cases where parental information was available either as trios or duos and excluding cases with structural variation that Exomiser cannot analyze. Note difference in scale of x-axis in panels A and B.

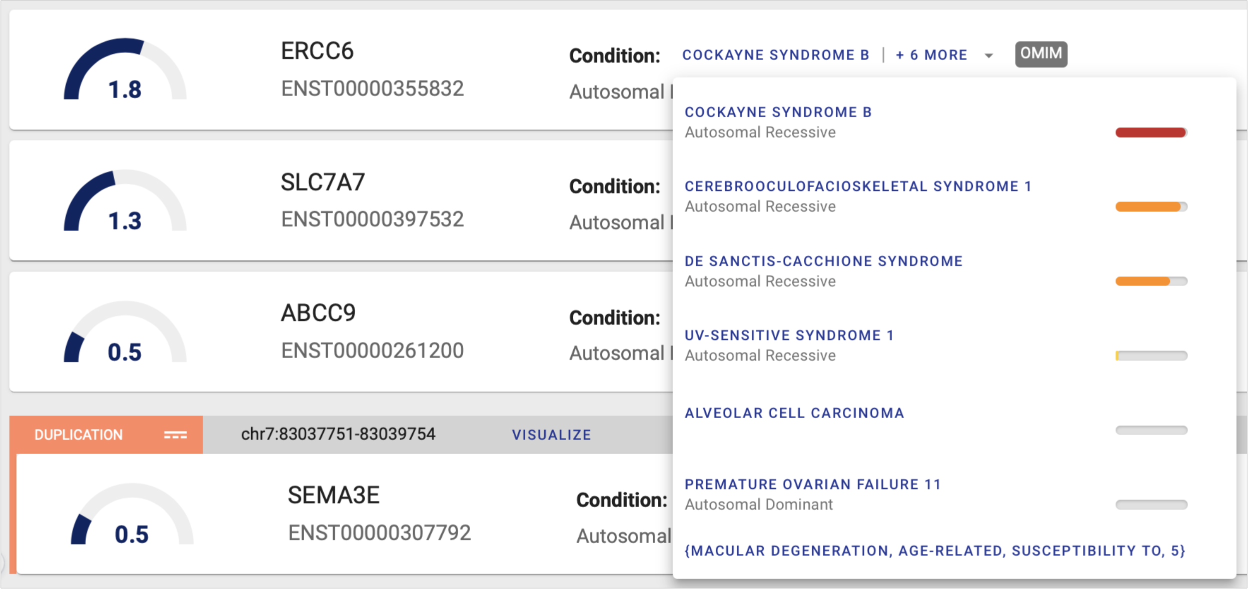


**Figure S7. Diagnostic nomination with condition match scores in GEM report.** The CM scores (color coded to represent relative magnitude and degree of Bayes Factor support) are shown next to each condition associated in a drop-down menu. The condition with the highest CM score is shown first and the remainder are sorted by the score in order of relevance as a candidate diagnosis. CM scores are normalized to 0-1 values and shown as color-coded bars next to each condition. Known mode(s) of inheritance for each condition are also noted and links to the OMIM database are provided for further information. For the case illustrated here, the gene ERCC6 was the highest ranked by GEM and is the true positive. ERCC6 is associated with 6 conditions with HPO profile and mode of inheritance, and a condition with no mode of inheritance described (not ranked by the CM score). From this display it is clear that the conditions at the bottom are poor fits to the patient phenotypes (CM scores, from the bottom: -1.42, -0.94, 0.19), whereas the top 3 can be diagnostic candidates (CM scores, from the top: 1.84, 1.46, 1.58) with GEM CM score favoring Cockayne syndrome B, the actual true positive diagnosis for this case.
